# Reconfiguring Rehabilitation Services for Rural South Africans with Disabilities During a Health Emergency: A Qualitative Descriptive Study

**DOI:** 10.3390/ijerph22040567

**Published:** 2025-04-04

**Authors:** Litakazi Tekula, Madri Engelbrecht, Lieketseng Ned

**Affiliations:** Division for Disability and Rehabilitation Studies, Department of Global Health, Faculty of Medicine and Health Sciences, Tygerberg Campus, Stellenbosch University, Room 4006, 4th Floor, Education Building, Cape Town 7505, South Africa; madrieng@sun.ac.za (M.E.); lieketseng@sun.ac.za (L.N.)

**Keywords:** orthotists and prosthetists, COVID-19, rehabilitation services, Eastern Cape, reconfiguration, healthcare, South Africa

## Abstract

The COVID-19 pandemic and the subsequent hard lockdown in South Africa, implemented in March 2020, significantly disrupted disability and rehabilitation services. Persons with disabilities experienced limited access to essential Orthotic and Prosthetic services, particularly in rural provinces such as the Eastern Cape. This study aimed to explore how Medical Orthotists and Prosthetists reconfigured their services during and after the pandemic to inform disability-inclusive emergency responses. A descriptive qualitative study was conducted with 12 Medical Orthotists and Prosthetists practicing in the public sector in the Eastern Cape. Semi-structured interviews were conducted via MS Teams, and the data were analysed by using thematic analysis to identify key themes related to service disruptions and adaptations. Four main themes emerged: (1) disrupted access to Orthotic and Prosthetic services, (2) backlogs and limited services, (3) safety measures and adaptation control, and (4) lingering challenges and gaps. Service delivery was hindered by halted outreach clinics, limited access to materials, budget reallocations, and the deprioritisation of rehabilitation services. This study highlights the challenges faced by Medical Orthotists and Prosthetists in maintaining the functionality of Orthotic and Prosthetic services during the pandemic. These findings emphasise the need for disability-inclusive policies and strategies to ensure the continuity of rehabilitation services during emergencies.

## 1. Introduction

The COVID-19 pandemic, declared a global health crisis in early 2020, created unprecedented challenges for healthcare systems worldwide. Prior to the pandemic, one billion individuals with disabilities experienced a severe lack of essential healthcare because persons with disabilities experience more difficulties in accessing healthcare than the general population [1]. The pandemic exacerbated pre-existing health disparities, disproportionately affecting disadvantaged populations, including persons with disabilities [2,3,4,5]. Such disparities are concerning because persons with disabilities often rely heavily on health and rehabilitation services [6], yet they faced aggravated barriers to accessing such services due to pandemic-related restrictions [7].

In South Africa, government-imposed lockdown measures and restrictions in March 2020 halted many healthcare services, including rehabilitation and assistive device-based services—services which were initially deemed non-essential [4]. Approximately 3.8 million people, or 7.5% of the population of South Africa, are reliant on these services [8]. COVID-19 regulations regarding healthcare services created a significant strain on public rehabilitation services, particularly for Medical Orthotists and Prosthetists (MOPs) who provide essential Orthotic and Prosthetic (O&P) support to individuals with disabilities. O&P services are essential to maintaining mobility, independence, and quality of life for persons with physical impairments who are in need of O&Ps. As these services were suspended or modified, persons with disabilities, who rely on these services, were faced with disrupted or halted routine services. While the number of service users increased during the COVID-19 pandemic, the support for service users was severely limited during hard lockdown [9].

### 1.1. COVID-19 and Rehabilitation Services

Globally, COVID-19 protocols led to the suspension of many services to prioritise COVID-19 treatment, despite the critical role these services play in supporting disadvantaged populations [10]. The pandemic’s impact on healthcare systems was particularly acute in regions where public health infrastructure was already under strain, such as in South Africa [11]. The result was a widening of health disparities, as those reliant on services like physical therapy, mental health support, and assistive technologies faced additional barriers to care [1,3,4]. Practitioners and facilities were often redirected to perform pandemic response activities, suspending rehabilitation services [12]. When persons with disabilities are unable to access routine rehabilitation services, their quality of life, mobility, functionality, and participation are negatively impacted. These impacts were more severe for those living in rural South Africa, where even more pronounced occupational inequalities and barriers to receiving necessary healthcare and rehabilitation services are routinely experienced [13]. The impact of these measures highlighted the urgent need for disability-inclusive policies and systems to ensure the continuous operation of essential services in times of crisis [2,12].

### 1.2. Experiences of Rehabilitation Practitioners During COVID-19

Rehabilitation practitioners similarly faced challenges that forced them to adjust their practices, with many undergoing additional training to support pandemic response initiatives, such as COVID-19 testing [14]. The provision of assistive equipment and on-going specialised treatment, such as wheelchair seating, were not always available, or prior authorisation was required for rehabilitation services [4]. MOPs, specifically, encountered unique barriers, such as Personal Protective Equipment (PPE) shortages and material supply issues, which further limited their capacity to provide essential services. Studies from India and the United Kingdom documented the operational changes made by O&P practitioners, including limiting face-to-face interactions, managing reduced patient loads, and adopting tele-rehabilitation for remote consultations [15].

Rehabilitation practitioners assisted in other health areas that were not part of rehabilitation. Van Biljon and Van Niekerk, and Adams et al. reported that while practitioners underwent additional training during COVID-19, they expressed concerns about practising outside the parameters of their scope of practice [14,16]. For example, rehabilitation practitioners assisted in swabbing tents where screening and triaging for COVID-19 were conducted [16]. They also had to work in COVID-19-dedicated wards with limited guidelines and protocols [17,18,19], while others offered mental health services as the demand increased, due to pandemic stress [20]. This meant that they could not serve their rehabilitation patients or they had to send them home because of the unavailability of staff and resources. Patients being discharged too soon, outpatient clinics closing, and outpatients arriving for rehabilitation appointments and being refused entry to the hospital were all issues that raised concerns for rehabilitation practitioners [14]. Practitioners had to reconfigure their services, presenting a challenge for their scope of practice during this time.

### 1.3. Knowledge Gap in Rural Rehabilitation Services

Little is known about how MOPs responded to the pandemic so as to include persons with disabilities in their service provision during the pandemic. One South African study documented the experiences of rehabilitation clinicians in Gauteng Province, a widely urbanised province [14]. Another study investigated the experiences of physiotherapists, occupational therapists, speech and language therapists, and audiologists in the public sector across South Africa, in diverse settings across the provinces and between urban and rural contexts [21]. However, none of these included MOPs. Limited data are available about COVID-19 responses in relation to O&P services in the Eastern Cape, one of the country’s most resource-constrained provinces.

This study sought to address this gap in evidence by providing an analysis of how MOPs in the Eastern Cape province adapted their practices, and, in addition, assessing the long-term implications for rehabilitation services in the region and potentially under future health emergency circumstances. Developing more efficient and disability-inclusive systems might benefit from research investigating the effects of COVID-19 from the perspective of service providers serving persons with disabilities [22]. The study thus aimed to explore how O&P services were reconfigured by MOPs in the Eastern Cape during and after the COVID-19 pandemic. The central research question guiding this investigation was as follows: How did Medical Orthotists and Prosthetists reconfigure their services in the Eastern Cape Province during and after the COVID-19 pandemic? By examining the strategies, challenges, and long-term effects of these adaptations, the study seeks to provide a nuanced understanding of the implications for healthcare policy and the future of O&P service delivery in times of health crisis.

## 2. Materials and Methods

This study adopted a descriptive qualitative approach to capture the direct experiences and perceptions of MOPs working in public hospitals in the Eastern Cape. This approach promotes the exploration of nuances inherent in service adaptation and documents the challenges faced by practitioners in their own words. The rationale for choosing a qualitative descriptive approach was to gain direct accounts of the phenomenon, as well as providing an analysis and interpretation of the findings that required reflective responses from participants [23]. This ensured that any information obtained was not overly synthesised but remained an accurate account of the words of the participants.

Qualitative descriptive studies play an important role in healthcare research by offering insights into service users’ experiences, and provider’s perceptions and experiences of certain occurrences, which can inform healthcare service provision [23]. This type of methodology is suitable for studies that are carried out in the health sector, as these involve patients who are recipients of public healthcare and health professionals who render services. It is crucial that service provision is continuously reviewed so as to ensure relevant service provision, upon which the majority of South Africans depend.

### 2.1. Study Setting

This study was conducted in Eastern Cape Province public hospitals offering O&P services. These hospitals are based in the bigger cities and service the smaller surrounding towns and remote areas. There are three public hospitals that offer O&P services in the province, namely, Bedford Orthopaedic Hospital in Mthatha, Frere Hospital in East London, and Port Elizabeth Provincial Hospital in Gqeberha. Bedford Hospital serves patients from OR Tambo District, while Frere Hospital serves patients from Amathole District. Port Elizabeth Provincial Hospital serves Sarah Baartman District. The first author conducted the study as part of her master’s degree, and the co-authors were her supervisors. The first author is also a Medical Orthotist and Prosthetist, while the co-authors are occupational therapists. The co-authors have more than 10 years of experience as qualitative researchers and have conducted similar research studies with other rehabilitation practitioners.

### 2.2. Study Population and Recruitment

**Figure 1:**
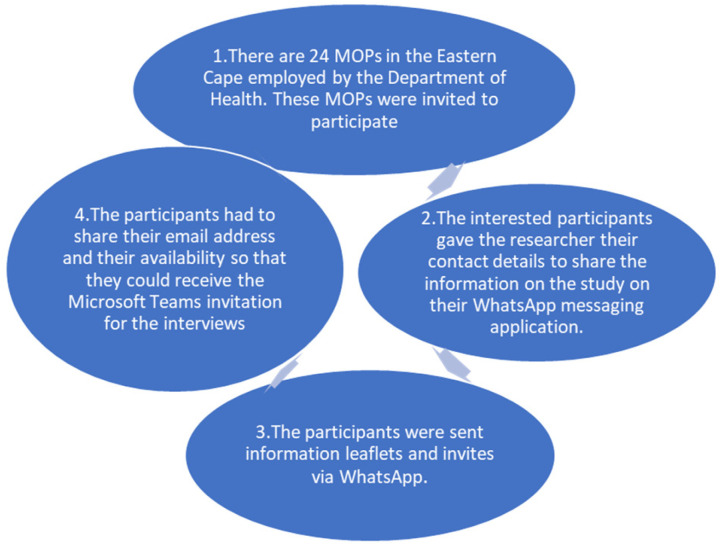
Recruitment process for participants.

The study population included all 24 MOPs who were employed in the public sector (Department of Health) in the Eastern Cape during and after the COVID-19 pandemic. The total population sampling strategy was used in this study. Total population sampling is performed when the target group is small and set apart by an uncommon and well-defined characteristic. Participants were first informed about the study in the MOPs’ quarterly forum meeting, and interested participants left their contact details along with an indication of their preferred method of communication (see Figure 1 below). Of the 24 MOPs employed in the Eastern Cape, only 12 agreed to participate in the study.

Most participants indicated that they preferred to communicate via the WhatsApp messaging application, and this platform was used to send invitations to them. Some participants added the researcher on their WhatsApp work group for communication with all members of their department. Only MOPs employed by the Department of Health prior to the pandemic and offering services during and after the COVID-19 pandemic were eligible for participation in the study. The participants were selected based on their experience of providing Orthotic and Prosthetic services before, during and after COVID-19. This ensured that data recorded during data collection reflected experiences during the COVID-19 pandemic and its aftermath in the Eastern Cape. MOPs who had retired or resigned after the pandemic were not included as participants. MOPs who were employed in 2023 as junior MOPs were also excluded, as they were new graduates without experience of working during the pandemic. The newly appointed MOPs were excluded for not having experience of working in a public sector prior to the pandemic.

Subsequent to ethics approval by Stellenbosch University, further approval for this research study was obtained from the Eastern Cape Department of Health. The approval was sent to the Heads of Departments to notify them of their employees’ involvement in the study. Following this, invitations to participate in the research study were sent to the 12 participants via WhatsApp. The invitation included an information sheet, accompanied by a consent form, which was signed before data collection started. Participants were given over a week to read the document before sending it back, and if they had any enquiries, they either phoned or sent a message via WhatsApp or an email to the investigator. Informed consent forms were signed online and returned via WhatsApp by some participants, while others sent an electronic signature, explaining that signing the document on their apps would have been difficult for them. Participants signed the consent form and returned it via email. Some participants could not print the forms, and they sent virtual signatures for the consent forms.

### 2.3. Data Collection

Data were collected through semi-structured interviews conducted on MS Teams by the first author. Semi-structured interviewing was chosen to foster a reciprocal relationship between the interviewer and participant, allowing the interviewer to create follow-up questions based on the participant responses and allowing for the participant’s unique speech expressions [24]. A semi-structured interview gives each individual interview naturally occurring conversational flow and structure [25]. The interviews were conducted online based on the availability of the participant’s schedule across the different centres as the researcher was unable to reach all the participants during working hours at the hospitals. The core questions that were addressed in the interview were the following: How were O&P influenced by the COVID-19 pandemic, and how were services influenced after the COVID-19 pandemic? Further probing questions were asked based on the responses. These interviews were conducted on the weekends at a time convenient for the participants. This platform provided flexibility in terms of scheduling and accessibility to participants. The initial interviews ranged from 25 to 40 min, and follow-up interviews were between 15 and 20 min on average. The initial interviews were conducted to obtain their initial insights. The follow-up interviews were conducted for clarity and to pose follow-up questions should any of those arise during transcription. They were also performed to conduct member checking with the participants. This was also to ensure that the correct data were transcribed and there was no omission. The total number of initial interviews conducted was 12, with 8 follow-up interviews. The four participants who did not participate in the follow-ups were unresponsive to requests. An interview schedule was developed and used to guide interviews, and questions prompted participants to discuss their experiences, challenges, and strategies in adapting their services during the pandemic.

All interviews were audio-recorded and transcribed verbatim by the first author. Transcripts were then read and member-checked to ensure the accurate capturing of participant insights. Data in the form of transcription documents were stored on a password-protected laptop. The audio-visual data of the interviews were stored in the secure Stellenbosch University OneDrive. Upon the completion of the transcriptions, we compared each transcript to its corresponding audio-visual format to ensure consistency of meaning. Interview files were saved with name and number sequences which corresponded to each participant’s pseudonym and interview number and the date of the interview.

### 2.4. Data Analysis

Thematic analysis was appropriate for this qualitative study. Data analysis was conducted by using the six-step guide to thematic analysis as described by Braun and Clarke (2006) [26]:

1. The first author acquainted herself with the data by listening to the audio-visual recordings of each interview on MS Teams. This process was repeated four times while checking against the transcripts for the accuracy of the data with the co-authors. The first author documented her thoughts in the form of notes and stored raw data of all notes, transcripts, and reflective notes.
2. The first author sorted and organised the data manually, grouping the data into codes by using tables on Microsoft Word. The codes were informed by words, phrases, and sentences that addressed the research question. These were shared with the co-authors.
3. The first author analysed the codes by using a reflexive diary with sub-themes and main themes. Codes were grouped into sub-themes. These sub-themes were grouped into themes. These codes were further compiled in a Microsoft Word document.
4. Themes and sub-themes were reviewed and analysed by the co-authors, who were supervisors of the first author.
5. The generated themes were then defined and named by all authors.
6. The findings were written in the form of themes and sub-themes with explanations supported by verbatim quotes from the participants, as presented in the Results section. The reflexive diary was used to make sense of the data and making notes on interpretation. This helped when writing up the Results and Discussion sections.

### 2.5. Ethical Considerations

The Stellenbosch University Health Research Ethics Committee, with HREC Ref No. S23/10/255, approved the research proposal. Once this permission was granted, further approval was obtained from the Provincial Department of Health. It was important that these institutional permissions were obtained so as to ensure the privacy of both the practitioners and patients and any information that might arise about patients that could violate the patients’ rights. During these applications, the interview guide and the informed consent form were included. Participants were compensated with vouchers and data bundles for some of the participants to join the interviews on MS Teams.

The following ethical principles were considered for this study: Beneficence, Confidentiality, Non-Maleficence, and Respect of persons (Dignity and Autonomy).

- Beneficence: Beneficence is the moral duty to maximise good and minimise bad [27]. We ensured that participants were well informed about risks that they might be exposed to and ensured that consent was granted at all times. Participants were made aware of the potential data risks that might happen should data be accessed by unauthorized personnel in the event of being hacked. This was mitigated by using pseudo-names on all stored data to protect the identity of the participants. The interviews were also saved in a different system in an encrypted folder.
- Non-Maleficence: All parties involved in the research study, including participants, participating communities, and the larger South African society, should be treated fairly in terms of risks and benefits [27]. Participants were informed about what was required from them and that their human rights would not be violated. Once the interviews were performed, participants were compensated with gift vouchers and data bundles to connect to the internet.
- Autonomy and Dignity: People’s decisions must be treated with respect, and they must be given the opportunity to exercise their right to self-determination [27]. Every participant had the right to express themselves in the interview, without infringement of their rights. We also ensured their privacy by safeguarding Confidentiality.

### 2.6. Ensuring Trustworthiness

The credibility of data and findings was ensured through the use of member checking during the follow-up interviews. Participants were sent invitations for an online session and presented with their transcribed interview to check and confirm that the data represented a true reflection of what they had shared. The researcher recorded supporting evidence consisting of direct quotes from the study participants, these being checked by the researcher’s supervisor to ensure that the findings accurately represented what was researched. Regular submissions were made to the supervisor and co-supervisor (co-authors) of the research study to manage subjectivity and bias on the part of the researcher. Dependability was ensured by creating an audit trail of detailed information, all revised information, and changes that were made in the research protocol. This ensured that no information was lost. Confirmability was improved by means of MS Teams and telephonic mode for member checking to ensure that the data were interpreted as closely as possible to the participants’ meaning and make any changes to the data to ensure accuracy. These safeguards ensure that the methodology employed in this study could be replicated under similar conditions as those presented by the global pandemic.

## 3. Results

### 3.1. Description of Participants

A total of 12 MOPs from Bedford Orthopedic Hospital (*n* = 5; OR Tambo District), Frere Hospital (*n* = 4; Amathole District), and Port Elizabeth Provincial Hospital (*n* = 3; Sarah Baartman District) participated in the study. Table 1 shows the demographics of the participants from the different hospitals. One of the participants was a Chief MOP, while the others were all grade 1 practitioners.

Four themes were developed from the data, namely, (1) disrupted access to O&P services, (2) O&P backlogs and limited services, (3) safety measures and adaptation, and (4) lingering challenges and gaps.

### 3.2. Themes Emerging from the Data

#### 3.2.1. Theme 1: Disrupted Access to O&P Services

The onset of the pandemic significantly disrupted access to O&P services across the Eastern Cape due to these services not being recognised as essential. The deprioritisation of services led to widespread confusion among practitioners, as Participant 10 noted:

“No-one knew what we were supposed to do. People in charge were not even aware that the O&P department existed or what we do”.

Patients, especially those from rural areas who needed assistive devices usually provided by MOPs, were severely affected by the cancellation of outreach clinics, limiting their access to these essential services. Due to the cessation of such outreach services, participants resorted to seeing only patients who were allowed to enter the hospital if they did not present with any COVID-19 symptoms. Participant 12, who worked in Sarah Baartman District and serves eight districts, stated the following:

I was concerned by the outreach clinics that were cancelled which meant that patients could not access these services because some do not have access to transport or cannot afford to pay for transport [to] come see us. It means that functionality will be decreased and we would suffer a lot because of this.

Most participants believed that outreach services would have aided accessibility, as there was limited access to transport that would bring patients to the hospital facilities. Participant 8 asserted the following:

“The continuation of outreach would have assisted in ensuring accessibility of services to people with disabilities”.

Moreover, hospitals differed in their responses to pandemic regulations, with some O&P centres revealing that they continued to operate, while others provided only limited services by, for example, excluding the provision of certain custom-made medical devices. Participant 7, from Amathole District, declared that their services were “One hundred percent operational”. Participant 11, from Sarah Baartman District, however, reported that some of their services were limited:

“Most services continued but casting and making new devices were only for those that were urgent”.

Some departments implemented an appointment system to control the number of patients seen per day. This led to a general decrease in patients accessing the service.

Conversely, in some departments, services continued as previously, as stated by Participant 8:

“Everything continued as normal like there was nothing new that was put in place to be guided by. So, we had to work as normal”.

Participant 6 reported yet another approach to appointments and services in their department:

“Patients were seen according to the number of patients that are accepted by OOPD [Orthopaedic Out Patient Department]. We receive walk-in patients from that department. We did not have our own schedule so we are relied on them”.

It was a concern for MOPs from Sarah Baartman District and Amathole District that the number of patients accessing the service decreased substantially due to the introduction of a new appointment system which allocated a specific number to be seen per day to avoid walk-ins and restrict numbers. Participant 8 from Amathole District reflected how patients’ decisions not to attend services similarly impacted the frequency of accessing services:

“Services were not halted but patients just did not show up and numbers decreased during this time. Everything was kept close to normal as possible”.

#### 3.2.2. Theme 2: O&P Backlog and Limited Services

The imposed pandemic restrictions on service delivery created a substantial backlog of patients waiting for new or replacement assistive devices. Budget reallocations toward COVID-19-related expenses, particularly PPE, further exacerbated resource constraints. Participant 9 summarised the impact as follows:

“We experienced a heavy backlog and limited assistive devices. Patients would not receive the appropriate rehabilitation services they needed”.

This was a common reality for O&P centres in the Eastern Cape. Participant 6 added how the redirecting of funds towards PPE purchases impacted O&P services:

“We could not buy certain materials for patients if the money was redirected to the purchase of PPE. This has resulted in us having a backlog and disadvantaging the patients with disability. It meant that [persons with disabilities] will further not receive treatment and this will negatively affect them”.

Participants were worried about having to control the number of patients seen per day and expressed their concern about limited access to O&P devices due to a lack of material.

There was, furthermore, a concern about the assistive devices that patients were receiving. Some participants indicated that there were only certain devices that they could issue in their hospitals, while others indicated that they were issuing them as per normal. Participant 12 stated the following:

“Most services continued, but casting and making new devices were only for those that were urgent. Patients that needed their devices repaired were prioritised during this time. New devices were not issued and this created a long backlog. Orthotic devices were prioritised so that we can avoid contractures. We also continued issuing all off-shelf devices”.

Participant 1 related how restrictions on numbers of patients limited the issuing of assistive devices:

“All services were rendered but a professional was only allowed to see a certain number [of patients] per day in order to reduce the risk of contracting the virus”.

Participant 7’s reflection highlighted the impact of COVID-19 on practitioners and the consequences for O&P services:

“We were one hundred percent operational. We did manufacture of orthotic and prosthetic devices. The only time we became a bit congested was when a staff member got ill or even staff members’ families got ill then they had to be out of work. But service continued the same”.

Most participants did indicate that although their departments were fully operational, patients did not access their services. This contributed to the backlog and resulted in strain on the services after the pandemic. Participant 1 (Bedford Hospital) explained that resource allocation to O&P services was not adapted after the pandemic to assist with backlogs:

“Nothing has changed post COVID-19 but we are trying our level best in decluttering the backlog it has left for us”.

According to most participants in this study, the backlog remains a common challenge for the Eastern Cape Orthotics and Prosthetics department. Participants highlighted the lack of consideration for disability-specific needs in the pandemic response, which contributed to delayed care and increased strain on O&P services. Participant 11 noted the following:

“There was little consideration for persons with disabilities … people could not access our services during this period, increasing the backlog”.

Participant 6 bemoaned the de-prioritisation of O&P services and the impact this had on clients with disabilities:

“O&P was an afterthought and we could not buy certain materials for patients if the money was not redirected to the purchase of PPE. This has resulted in us having a backlog and disadvantaging the patients with disability. It meant that persons with disabilities will further not receive treatment and this will negatively affect them”.

Participants reported that the unavailability of transport for patients impacted their access to O&P services during COVID-19. A participant from Amatole District relayed the following:

“A certain number of patients were taken a day, this was done so as to avoid a huge number of patients coming to the hospital and risk of contracting the virus”.

Most participants from the different hospitals mentioned that they did try to keep service delivery as close to normal as possible; however, patients were not attending because of different factors, which included financial or social constraints.

#### 3.2.3. Theme 3: Safety Measures and Adaptation Control

To mitigate infection risks, practitioners implemented appointment systems and limited the number of patients seen daily. Physical distancing and sanitisation protocols were adopted, with staff working alternate shifts to reduce exposure. Participant 7 remarked the following:

“We implemented our own infection control measures … like hand sanitisers and social distancing”.

Despite these efforts, practitioners faced the constant challenge of balancing patient care with their own health and safety, often improvising due to a lack of centralised guidelines, as expressed by Participant 10:

“We had to come up with what we can do or cannot do to make sure that we don’t get infected or infect the patients”.

The appointment system was aimed at managing the flow and volume of patients who attended services daily in order to reduce the risk of infection.

Participants were motivated to help patients but had to also protect themselves from the virus. Fear of infection increased their anxiety, as some participants became infected even though safeguarding measures were in place at work. Participant 4 (Bedford Hospital) mentioned the following:

“Due to small space of work we were instructed as staff members to rotate, and fifty percent of the staff be at home and fifty percent be at work. However, if you are at home, you should consider yourself as someone who is on standby. Should the need arise for you to be at work you should avail yourself. If not, you sign leave. This was to ensure the continuity of the services to the public but at the same time adhere to the rules and guidelines of the pandemic”.

The O&P centre in Sarah Baartman District operated according to a similar principle, as explained by Participant 11:

We alternated, the staff member would leave earlier in one particular instance on this date and the other one on the next day. They also had days where they fumigated the place when everyone had to leave should there be a person that tests positive for COVID-19.

Although there were some differences in the approaches by different centres, strategies utilised were largely similar and had the same intention, which was to decrease the number of staff on duty at the same time.

Participants reported the formation of committees that were designed to ensure that the spread of COVID-19 was curbed while full service delivery continued, taking into consideration the likelihood of future pandemics. Participant 4 explained this as follows:

“Staff attended some trainings conducted by infection control manager about the pandemic to be equipped and be ready for any pandemic that might come in the future”.

While strategies were implemented for infection control during and after the pandemic, not all hospitals developed specific COVID-19-related strategies. The implementation of committees was to ensure that there was preparedness and that infection control could be adhered to.

#### 3.2.4. Theme 4: Lingering Challenges and Gaps

The pandemic’s impact lingers as practitioners contend with material shortages, staffing issues, and a patient backlog post-COVID-19. Participants indicated that the aftermath of COVID-19 can be felt the most when it comes to the issuing of devices, as a result of a number of factors. Participant 3 (Bedford Hospital) relayed how the challenges of working during COVID-19 prevailed after the pandemic:

“My concerns were shortage of materials, patients not coming to the hospital for services as told or on appointments, not being able to deliver to patients to our fullest potential because of the fear of the unknown following the pandemic. Even after the pandemic none of the concerns have changed because we are still in the same situation as compared to that time of the pandemic”.

Participant 10 from Amathole District stated the following:

“The COVID-19 pandemic helped give us recognition as essential services and this meant that patients could still access our services regardless of some of the restrictions on services that were rendered during this time”.

There is little to no knowledge of the scope of practice of MOPs. This has assisted in improving the allocation of resources to the department, which will assist in service delivery in the Eastern Cape. The MOP professionals felt that there is a growing need to consider future strategies for ensuring preparedness in future pandemics. Participants from Sarah Baartman District did mention that innovative strategies and committees need to be sustainable so that there preparedness for future outbreaks can be achieved.

## 4. Discussion

The findings of this study reveal that MOPs in the rural Eastern Cape Province encountered significant challenges in maintaining service delivery during the COVID-19 pandemic. Their experience is consistent with international reports on the impact of COVID-19 on rehabilitation services [15]. The designation of O&P services as non-essential under pandemic circumstances highlights a systemic oversight that left disadvantaged populations underserved and practitioners without clear guidance.

McKinney et al. (2021) [2] reported that under lockdown regulations, access to rehabilitation services (including assistive technology) and specialised care in South Africa was restricted or suspended. Rehabilitation practitioners explained how they adjusted staffing levels to control social distancing in order to safeguard clinicians and patients in the clinical setting [15]. Respondents in this study affirmed how they continued to operate in the absence of specific service directives. As a result, they had to implement their own infection control measures that would ensure protection for both patients and practitioners. In addition, they implemented appointment systems that would assist in ensuring accessibility to services while controlling the number of patients seen.

In a study by Fernandes and Donovan-Hall (2024), rehabilitation professionals discussed how relocating Prosthetics therapy, restricting face-to-face interactions, and managing the patient workload with fewer staff members were among the essential adjustments made to their services during COVID-19 [28]. Numerous participants in Puli et al.’s [15] study explained that they took significant steps to decentralise their services from hospitals or institutions to the people. Conversely, participants in this study reported how services were reconfigured, resulting in outreach clinics being discontinued and a restricted effect on service access for persons with disabilities. Participants also stated that they prioritised Orthotic devices that were regarded as urgent and that new Prosthetic devices were thus not issued. To replace some of the services that had become restricted during the pandemic, Van Biljon and Van Niekerk [14] recommended tele-health as a viable alternative to face-to-face intervention under health emergent circumstances. In another study in Gauteng Province, South Africa, practitioners stated that there were no clear guidelines on how to incorporate tele-health [20]. The viability and practicality of tele-rehabilitation in the context of the current study is questionable due to lack of infrastructure and the socio-economic challenges of persons with disabilities from rural Eastern Cape areas.

Prior to the pandemic, persons with disabilities in Africa faced obstacles to accessing health and rehabilitation services related to transportation and travelling distances, expenses, waiting times, and the physical reach of establishments, among other issues, culminating in a reduced total consumption of healthcare services [29]. The findings of the current study confirm that COVID-19 restrictions exacerbated these existing issues, particularly affecting O&P services, which experienced severe backlogging. The reduction in rehabilitation budgets further hampered the production of assistive devices, intensifying these backlogs.

Additionally, transportation difficulties, compounded by COVID-19 restrictions, made it even more difficult for patients to access healthcare. Puli et al.’s study noted that when transportation challenges hindered patients’ attendance of rehabilitation sessions, assistive technology practitioners often went above and beyond to help patients attend service settings [15]. Smith et al. [30] also highlighted that the public health measures implemented in response to COVID-19 further reduced the functionality of the system linked to public health reaction to COVID-19, preventing individuals with pre-existing conditions from receiving the necessary assistive devices.

South Africa’s lack of preparedness and resource constraints mirrored these global trends in low- and middle-income countries, such as Peru or Yemen, where systemic weaknesses exacerbated health disparities. For instance, Carta et al. [31], compared responses of health systems to the pandemic by using cases reports from the United States, Germany, Vietnam, New Zealand, Cuba, and Italy, and this comparison underscores how systemic inequalities in healthcare systems influenced pandemic outcomes, particularly in vulnerable populations. Such findings could be compared to the challenges faced by South Africa’s Orthotic and Prosthetic services with those of other countries with underfunded or fragmented health systems. Carla et al.’s paper also identifies good practices from countries like New Zealand (on collaborative governance) and Vietnam (on strict containment measures). These practices, such as centralized decision making or public engagement, could inform more effective disability-inclusive policies in South Africa. Adopting proactive measures such as prioritizing O&P services as essential could mitigate service disruptions during future emergencies. A participant of this study remarked that if O&P services had been designated as essential during the pandemic, the exacerbation of an already heavy service backlog and the resultant increased strain on O&P services would have been avoided.

### 4.1. Implications for Policy and Practice

This study emphasises the need to designate O&P as an essential service, especially during health emergencies. Such a designation would ensure continued access for disadvantaged populations and provide clear guidelines for practitioners, potentially reducing service backlogs and unmet needs. Developing clear protocols for infection control, staffing adjustments, and patient management would support practitioners in delivering safe, effective services during future health crises.

Additionally, there is a pressing need for policies that allocate sufficient resources and develop contingency plans for rehabilitation services, including funding for outreach and tele-rehabilitation options. Policy should guide investment in tele-health infrastructure, particularly in underserved areas, and provide training for practitioners to implement tele-health effectively. Addressing digital access issues for persons with disabilities is, therefore, also pertinent. Policy should further support the effective decentralisation of services to enhance accessibility in rural areas, through the provision of outreach clinics and mobile units in remote locations. This will promote better continuity of care in times of restricted movement. Finally, clear prioritisation criteria should be developed for the provision of assistive devices during emergencies. This will aid the effective management of resources and ensure that the most critical needs are met while simultaneously planning for the timeous resumption of full services.

Tele-rehabilitation in Orthotics and Prosthetics can be considered a potential platform for follow-up services, should government invest in resourcing its facilities with the necessary technological resources. The training of MOPs on the use of tele-health technologies and O&P care would be beneficial for tele-health to work. The use of 3D printing portable devices for Orthotics would be essential in remote areas to reduce the high backlog in O&P centres. However, patient education on using these services would be needed, with both advantages and disadvantages. Likewise, connectivity in rural areas would need to be improved.

### 4.2. Limitations

This study’s sample size was limited to 12 practitioners and was conducted remotely, restricting the depth of data collection. Linked to this, conducting interviews via MS Teams may have restricted rapport building and the depth of responses compared with in-person interviews. Future research should expand the sample size and explore the experiences of these professionals across the nine provinces of South Africa to capture a more comprehensive view of the pandemic’s impact on service delivery.

## 5. Conclusions

This study demonstrates the critical role of MOPs in supporting individuals with disabilities, especially during times of crisis. The experiences of MOPs in the Eastern Cape reveal systemic barriers to service continuity and underscore the importance of policy reforms that prioritise disability-inclusive healthcare. Future public health crises should consider rehabilitation services to be essential, accompanied by preparedness plans that facilitate resource allocation and service continuity for marginalised groups.

## Figures and Tables

**Table 1:** MOP practitioners’ district of employment and rankings.

Participant	Municipal District	Years of Experience	Gender	Position
Participant 1	OR Tambo District	3	Female	Grade 1 MOP
Participant 2	OR Tambo District	4	Female	Grade 1 MOP
Participant 3	OR Tambo District	3	Female	Grade1 MOP
Participant 4	OR Tambo District	4	Female	Grade 1 MOP
Participant 5	OR Tambo District	3	Female	Grade 1 MOP
Participant 6	Amathole District	6	Female	Grade 1 MOP
Participant 7	Amathole District	6	Female	Chief MOP
Participant 8	Amathole District	5	Male	Grade 1 MOP
Participant 9	Amathole District	5	Male	Grade 1 MOP
Participant 10	Sarah Baartman District	10	Female	Grade 1 MOP
Participant 11	Sarah Baartman District	8	Female	Grade 1 MOP
Participant 12	Sarah Baartman District	4	Female	Grade 1 MOP

## Data Availability

Data are contained within the article.
